# The Impact of the Final Sintering Temperature on the Microstructure and Dielectric Properties of Ba_0.75_Ca_0.25_TiO_3_ Perovskite Ceramics

**DOI:** 10.3390/ma17215210

**Published:** 2024-10-25

**Authors:** Kamil Feliksik, Małgorzata Adamczyk-Habrajska, Jolanta Makowska, Joanna A. Bartkowska, Tomasz Pikula, Rafał Panek, Oliwia Starczewska

**Affiliations:** 1 Faculty of Science and Technology, Institute of Materials Engineering, University of Silesia, 75 Pułku Piechoty 1A, 41-500 Chorzow, Poland; kamil.feliksik@us.edu.pl (K.F.); malgorzata.adamczyk-habrajska@us.edu.pl (M.A.-H.); joanna.bartkowska@us.edu.pl (J.A.B.); oliwia.starczewska@us.edu.pl (O.S.); 2 Institute of Electronics and Information Technology, University of Technology, 38A Nadbystrzycka Str., 20-618 Lublin, Poland; t.pikula@pollub.pl; 3 Department of Construction Materials Engineering and Geoengineering, Lublin University of Technology, 40 Nadbystrzycka Str., 20-618 Lublin, Poland; r.panek@pollub.pl

**Keywords:** Ba_0.75_Ca_0.25_TiO_3_ ceramics, structure, microstructure, dielectric properties, thermal analysis, pyroelectric currents, perovskites

## Abstract

Ba_0.75_Ca_0.25_TiO_3_ ceramics were successfully synthesized by a simple solid-state reaction method. This study examined the influence of sintering temperature on the structure, microstructure, dielectric properties and electrical behavior of the material. The XRD analysis reveals that the tetragonal phase (P4mm) is dominant in all the synthesized materials, with those sintered at *T* = 1400 °C and *T* = 1450 °C being single-phase, while others exhibit a minor orthorhombic phase (Pbnm). Higher sintering temperatures promoted better grain boundary formation and larger grain sizes. The electric permittivity increased with temperature up to *T* = 1400 °C, followed by a sharp decline at *T* = 1450 °C. Additionally, the Curie temperature decreased with increasing sintering temperature, indicating changes in phase transition characteristics. Thermal analysis showed that higher sintering temperatures led to sharper heat capacity peaks, while pyroelectric and thermally stimulated depolarization currents were maximized at *T* = 1400 °C due to oxygen vacancies. These findings highlight the significant impact of sintering temperature on the material’s structural and functional properties.

## 1. Introduction

Perovskites are a broad group of materials that are constantly attracting great interest due to their strong ferroelectric properties and piezoelectric and pyroelectric behavior and the possibility of influencing these properties by appropriately doping the base material and selecting appropriate technological process conditions. The perovskite family is described by a general formula of the form ABO_3_, in which the A and B positions are usually occupied by metal ions. The flagship representatives of ferroelectric perovskites include barium titanate, BaTiO_3_ (BT). Barium titanate is a ferroelectric compound that has a high dielectric constant and low dielectric loss. Therefore, it is one of the most widely used ceramic materials in electronic equipment [1,2]. The key applications of BT-type ceramics include positive-temperature-coefficient devices, pulse generators, infrared detectors, multilayer ceramic capacitors, voltage-tuning devices in microwave electronics, piezoelectric actuators, transducers, and charge storage devices [3,4,5,6,7,8]. These materials continue to attract significant interest not only for their properties but also because they are lead-free and environmentally friendly. This contrasts with popular Pb(Zr,Ti)O_3_ ceramics, which are toxic due to their lead content [9,10,11,12].

Undoped barium titanate is historically the first lead-free ferroelectric and piezoelectric material. In order to improve its physical properties, attempts were made to modify it with ions of various elements. For example, in order to improve piezoelectric properties, grain size control was started by modifying BT with dopant ions such as Ca, Zr, Sn and Hf. Undoped BT ceramic material showed piezoelectric coefficient d33 values at the level of 338–519 pC/N. However, after modification with dopant ions, the piezoelectric coefficient d33 value increased to 625 pC/N [7,13,14].

Many studies have been carried out to improve the dielectric properties of barium titanate. These studies have involved substituting different ions at the A or B positions in the perovskite structure of BT ceramics [15]. Namely, for example, P Wang et al. doped BT ceramic material with calcium and zirconium ions. Calcium ions were substituted at the A position, while zirconium ions were substituted at the B position in the ABO_3_ perovskite structure. The final ceramic material (Ba_0.85_Ca_0.15_(Ti_0.9_Zr_0.1_)O_3_ (BCTZ)) was obtained by the conventional method of mixing oxides at different calcination and sintering temperatures. By selecting the dopant ions and selecting the appropriate technological conditions, the authors obtained a ceramic material that is a very promising lead-free material of importance for a multitude of applications. The authors showed that the BCTZ ceramics, which were calcined at *T* = 1300 °C and sintered at *T* = 1540 °C, exhibited optimal electrical properties, i.e., d_33_ = 650 pC/N, d_31_ = 74 pC/N, k_p_ = 0.53, k_t_ = 0.38, k_31_ = 0.309, ε_r_ = 4500 and P_r_ = 11.69 μC/cm^2^ [16].

BT is characterized by its piezoelectric properties having a high dependence on temperature. In order to reduce this dependence and stabilize the piezoelectric properties, P. Zheng et al. attempted to dope BaTiO_3_ with Zr ions, which were substituted for Ti ions. They obtained a ceramic material, BaTi_0.9625_Zr_0.0375_O_3_, which was then modified with a small amount of CuO. The ceramic material obtained in this way has excellent piezoelectric properties, i.e., a piezoelectric coefficient of d_33_ = 300 pC/N, k_p_ = 0.493 and k_33_ = 0.651 with a tangent = 0.011, and a kp greater than 0.40 in a wide temperature range, namely from *T* = 43 °C to *T* = 73 °C, and its properties are almost constant in the range from *T* = 25 °C to *T* = 55 °C. The results from the cited authors indicate that the CuOBaTi, ZrO_3_-modified ceramics obtained by them are a promising lead-free material for practical applications, and appropriate doping significantly improves the properties of the tested material [17]. In turn, the work of A. Rached et al. presents the results of structural and electrical studies performed for barium titanate doped with erbium. The ceramic material Ba_0.91_Er_0.1/3_Ca_0.04_Ti_0.92_Sn_0.08_O_3_ was obtained by the solid-state diffusion method. The obtained material is characterized by a polycrystalline microstructure, as shown by SEM studies. Electrical conductivity studies allowed the determination of the activation energy, which is in the range from 1.3 eV to 0.213 eV and is dependent on temperature. Nyquist plots created for the obtained ceramics suggest that that the electrical properties of this compound are influenced by grain effects. The dielectric measurements performed indicate a high value of electric permittivity and reveal a phase transition from the ferroelectric to the paraelectric phase. Thanks to such doping, it was possible to increase the multifunctionality of barium titanate by improving its physical properties [18]. V. Paunovic et al. investigated the impact of Nb^5+^ on the microstructure and dielectric properties of BaTiO_3_ ceramics, using the solid-state method to produce Nb/Mn–BaTiO_3_. They found that increasing the Nb^5+^ content reduces the electric permittivity, with the highest values observed for 0.1 Nb/Mn–BaTiO_3_ sintered at *T* = 1350 °C. A higher Nb content led to lower electric permittivity and loss tangent values, as well as a decrease in the Curie constant. The optimal dielectric properties were observed in ceramics doped with 0.1 at% Nb, and the effect of Nb^5+^ depended on the sintering temperature [19].

Researchers have doped barium titanate with rare earth ions like La^3+^, Gd_3+_, Dy^3+^ and Yb^3+^ to improve its electrical properties. Doping with Sr^2+^ and Ca^2+^ increases piezoelectricity and electrical breakdown strength. On the other hand, doping with rare earth ions lowers the phase transition temperature and affects electrical properties. Lanthanum and gadolinium improve electrical performance, while dysprosium and ytterbium worsen it [20,21,22,23,24,25,26,27]. BaTiO_3_ is an excellent ferroelectric material, but by appropriate doping with iron ions it can be enriched with magnetic properties. The work of S.B. Bhoobas et al. presents interesting results from research on iron-modified barium titanate, i.e., FeBaTiO_3_. The authors report that barium titanate doped in this way retains its crystallinity in the tetragonal phase with the P4mm space group, with only a small change in the tetragonality ratio, the value of which changes from 1.008 (BaTiO_3_) to 1.002 (FeBaTiO_3_). The ferroelectric–paraelectric phase transition temperature for the modified FeBaTiO_3_ was shifted from 120 °C (for undoped BaTiO_3_) to 300 °C (for modified FeBaTiO_3_). Such properties suggest that this material can be used for high-temperature capacitive applications. Magnetic hysteresis (M-H) studies at room temperature performed on modified FeBaTiO_3_ show ferromagnetic properties [28,29,30].

Among so many modifiers, it is difficult to choose the most optimal one. An analysis of the literature data has shown that the most comprehensive benefits can be achieved by doping barium titanate with calcium ions. Primarily, by improving sinterability, they influence the stabilization of the crystal structure [31,32]. Additionally, Ca^2+^ ion doping leads to a significant reduction in dielectric losses while simultaneously increasing electric permittivity, which is advantageous for applications such as ceramic capacitors [33]. In these applications, the improvement in dielectric breakdown strength is also important, which is ensured by Ca^2+^ doping. Moreover, doping with calcium ions enhances piezoelectric properties [34], which significantly expands the application potential of BaTiO_3_-based materials.

Literature reports show how much influence the selection of the appropriate type and amount of BaTiO_3_-modifying ions has on the piezoelectric and electrical properties and their stability. The selection of appropriate technological conditions, including sintering temperature, also has a significant influence. That is, electrical properties, similarly to piezoelectric properties, can be controlled by introducing appropriate doping ions into the BT base material and strictly controlling both the chemical composition and conditions during the technological process [35,36,37,38,39,40,41,42,43,44].

There are several methods for obtaining BT ceramic material, both undoped and doped. These include the sol–gel method [1,45,46,47,48], spark plasma sintering [49,50] and solid-state synthesis. Due to the fact that the ceramic material presented in this paper may have great application significance, the authors used the solid-state synthesis method to produce it. This method, unlike other methods, allows for the production of ceramic material on an industrial scale and is an economical method.

Based on the analysis of literature data and the analysis of factors influencing the electrical properties of the modified BT ceramics, an attempt was made to dope BaTiO_3_ with calcium ions and investigate the effect of the sintering temperature on the properties of these modified ceramics. The aim of this work was to obtain Ba_0.75_Ca_0.25_TiO_3_ ceramic material and to investigate the effect of sintering temperature on the structural, microstructural, and dielectric properties. The tested Ba_0.75_Ca_0.25_TiO_3_ ceramic material was prepared using a mixed oxide and carbon method and synthesized by the free sintering method.

## 2. Materials and Methods

Ba_0.75_Ca_0.25_TiO_3_ ceramics were synthesized using barium carbonate (BaCO_3_, Avantor (Radnor Township, PA, USA, 99.99%), calcium carbonate (CaCO_3_, Avantor (Radnor Township, PA, USA), 99.99%) and titanium oxide (TiO_2_, Avantor (Radnor Township, PA, USA, 99.99%) as starting materials. The oxide and carbonate powders were first weighed in stoichiometric proportions, then mixed in a mortar before being further processed in a planetary ball mill. The wet milling process used ethanol (Avantor Radnor Township, PA, USA, 99.99%) as a solvent and zirconium–yttrium grinding media (diameter d = 1 cm) in polyamide containers for a duration of *t* = 24 h. After milling, the mixture was air-dried, followed by calcination of the dried powders in a muffle furnace at *T* = 1000 °C for *t* = 3 h. During synthesis, the furnace heating rate was set to *v* = 5 °C/min. The programmable temperature control ensured a linear temperature increase and stable temperature maintenance. The cooling rate of the synthesized samples corresponded to the free cooling rate of the furnace.

The synthesized powders were first mixed in a mortar, followed by wet milling in a planetary ball mill using ethanol as the milling medium. Zirconium–yttrium grinding media with a diameter of 1 cm were employed, and the process was conducted in polyamide containers for a duration of *t* = 24 h. After milling, the powders were air-dried. The dried powders were then pressed into compacts with a diameter of 10 mm using a semi-automatic hydraulic press at a pressure of *p* = 300 MPa in steel dies. These compacts were subsequently placed in corundum crucibles on a bed of alumina (Al_2_O_3_, Avantor, 99.99%) for further processing. The compacts were sintered in an electric resistance furnace at various temperatures—*T*_S_ = 1250 °C, *T*_S_ = 1300 °C, *T*_S_ = 1350 °C, *T*_S_ = 1400 °C and *T*_S_ = 1450 °C—for *t* = 3 h, in an air atmosphere. For the sintering process, the heating rate was the same as during synthesis. The samples for dielectric measurements were prepared in the form of disks with a thickness of *d* = 0.6 mm and a surface area of 1 cm^2^, with parallel surfaces coated with conductive silver paste (P-120, supplied by the Polish State Mint, Warsaw, Poland) using the burn-in method.

The phase composition of the prepared samples was evaluated using X-ray diffraction (XRD) on a Panalytical X’pert PRO MPD diffractometer (Eindhoven, The Netherlands) in the standard θ–2θ configuration. The diffractometer was equipped with a PW 3050 goniometer, a PIXcel 1D detector, and a copper (Cu) radiation source (CuKα = 1.54178 Å). For phase and structural analysis, the HighScore Plus 3.0e software was used, incorporating the 2022 edition of the PDF-2 DL database from JCPDS-ICDD.

The Archimedes method with distilled water was employed to evaluate the sample density. The microstructure of the produced ceramic samples, along with qualitative and quantitative EDS analysis, was examined using a JEOL JSM-7100F TTL LV scanning electron microscope (Akishima, Japan) equipped with an EDS system.

The dielectric properties were measured on specially prepared samples using a computerized system that included an Agilent E4980A LCR meter (Santa Clara, CA, USA). To reduce stresses from prior mechanical processing and allow for the relaxation of immobile defects formed during sintering, the samples were pre-heated at *T* = 400 °C for *t* = 30 min prior to measurements (heating rate *v* = 5 °C/min).

The enthalpy and characteristic temperatures of the ceramic samples were determined using differential scanning calorimetry (DSC1, Mettler Toledo, Columbus, OH, USA). BCT25 ceramic powders were weighed with an analytical balance (RADWAG AS 60/220/C/2) and encapsulated in 40 µL hermetically sealed aluminum crucibles. Measurements were conducted from room temperature to *T* = 300° C at a heating rate of *T* = 10 °C/min, using nitrogen as the drying gas. Results were analyzed with STARe software (Version 11.00a).

The temperature dependencies of pyroelectric currents and thermally stimulated depolarization (TSD) currents were measured using a computerized system with a Keithley 6485 picoammeter (Cleveland, OH, USA). Before the measurement, the samples were polarized in a DC field at *T* = 150 °C for 20 min and then cooled to room temperature in this field. The samples were then heated at a rate of 5 K/min up to 773 K, and the current was measured as a function of temperature and time.

## 3. Results and Discussion

### 3.1. X-Ray Analysis

**Figure 1:**
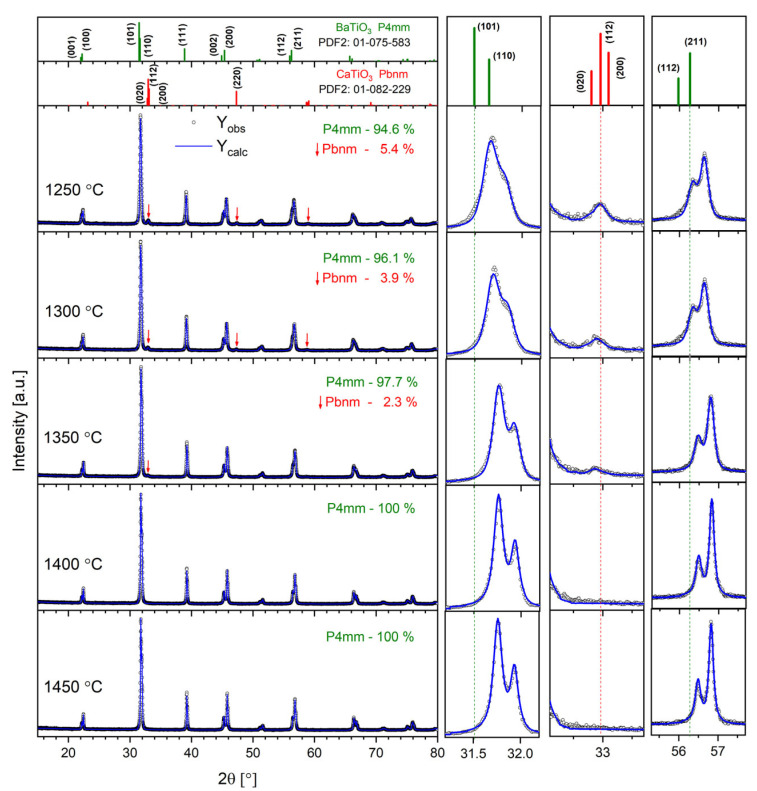
The results of XRD investigations for all the BCT samples studied. The right panel shows the magnification of the diffractograms in the 2θ ranges of 31–32°, 32–32.5° and 55–58°. The top panel shows standard patterns for tetragonal BaTiO_3_ (PDF2 card number 01-075-583) and orthorhombic CaTiO_3_ (PDF2 card number 01-082-229) as a reference.

Figure 1 shows the XRD patterns registered for all the studied Ba_0.75_Ca_0.25_TiO_3_ samples. It can be noted that the tetragonal phase (space group P4mm), characteristic of BaTiO_3_ at room temperature, dominates.

Ceramics sintered at *T*_S_ = 1400 °C and *T*_S_ = 1450 °C are single-phase, while a small amount of orthorhombic-phase ceramics (Pbnm space group), characteristic of CaTiO_3_ perovskite, was detected for the other samples. The top panel of Figure 1 shows standard XRD patterns for the tetragonal BaTiO_3_ and orthorhombic CaTiO_3_ as a reference. Thus, it can be assumed that only the highest temperatures, *T* = 1400 °C and *T* = 1450 °C, are sufficient to dissolve all the Ca^2+^ ions in the BTO lattice. On the other hand, a synthesis temperature of up to *T* = 1350 °C provides too little energy, and the Ca-rich orthorhombic phase is separated. Moreover, the contribution of the Pbnm phase gradually decreases with the growth of the sintering temperature, confirming the above findings. In turn, the volume of the Pbnm unit cell significantly increases with the rise in synthesis temperature, proving that some amount of Ba^2+^ ions replace Ca^2+^ ions in the orthorhombic Pbnm structure. To obtain the structural parameters, the Rietveld refinement method was applied. The results are displayed in Table 1. One can note the gradual decrease in lattice parameters with the increase in annealing temperature for the P4mm majority phase. This is mostly because more Ca^2+^ ions are located inside the BTO structure when the annealing temperature increases. The latter can be evidenced by the gradual shifting of diffraction peaks towards the higher 2θ angle visible in the right panel of Figure 1. For ceramics sintered at *T*_S_ = 1400 °C and *T*_S_ = 1450 °C, the lattice parameters are almost identical, as all the Ca has been dissolved in the BTO lattice. It is clearly visible in the right panel of Figure 1 that the width of the diffraction peaks gradually decreases with the increase in annealing temperature. This results from the lower level of lattice strains and imperfections caused by the elevated temperature.

### 3.2. Density and Microstructure

In the first stage of the research, the real density of the samples was determined using the Archimedes method (Table 2). The density of the sample sintered at the lowest temperature had the smallest value of ρ= 4.47 g/cm^3^, while for the material sintered at *T*_S_ = 1400 °C, this value was the highest, at ρ= 5.67 g/cm^3^. It is also worth noting that the density of the ceramic material sintered at *T*_S_ = 1450 °C was slightly lower, at ρ= 5.47 g/cm^3^.

**Figure 2:**
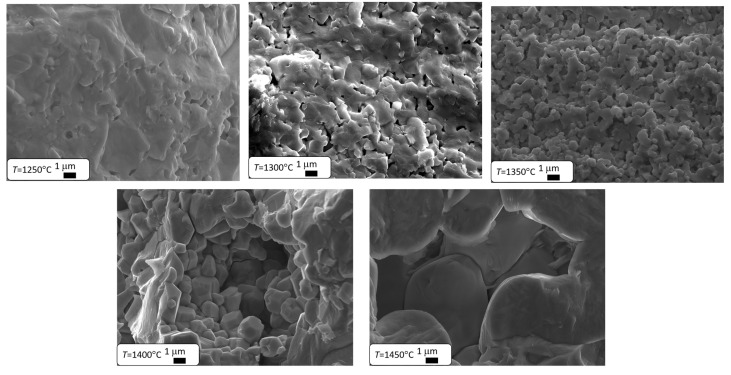
SEM images of Ba_0.75_Ca_0.25_TiO_3_ ceramics sintered at different temperatures.

The results presented in Table 2 clearly indicate that as the final sintering temperature increases, the material’s density significantly increases, which leads to a reduction in its porosity through diffusion kinetics [51]. The pores located at the grain boundaries significantly hinder their movement, which slows down grain growth. Once they are eliminated, grain growth becomes faster [1,41,42,43,44,45,46,47,48,49,50,51,52]. Therefore, it can be expected that increasing the final sintering temperature will lead to an increase in grain size. This theory was confirmed by microstructural studies conducted using a scanning electron microscope. Microstructure images of the ceramic materials discussed are shown in Figure 2. Similar results were observed in BZT-BCT 50/50 ceramics [53], where the authors explained the increase in grain size and reduction in pore size with rising sintering temperatures using the phenomenological kinetic grain growth equation, which suggests that grain size increases as sintering temperature rises [54].

In the case of the two lowest temperatures, the grain structure did not form. The grain structure only develops in ceramics sintered at *T*_S_ = 1350 °C. Further increases in sintering temperature lead to a better formation of grain boundaries and a significant increase in grain size (Table 2). A similar grain growth caused by an increase in sintering temperature is observed in Ba_0.70_Ca_0.30_TiO_3_ ceramics [55], as well as in many other materials based on barium titanate, such as BaZr_0.2_Ti_0.8_O_3_ [56] or BZT-BCT 50-50 [53]. The main factor behind grain growth is the decrease in the total grain boundary area, which lowers the interfacial free energy of the material [57]. Since interfacial energy is inversely related to grain size [58], this leads to a reduction in the overall grain boundary area. As the grains grow larger, the porosity of the structure decreases [59].

### 3.3. Dielectric Properties

**Figure 3:**
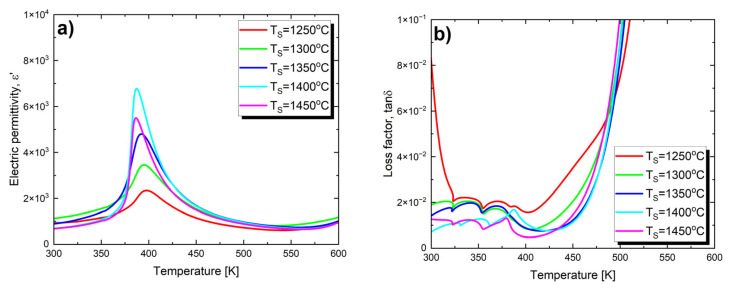
Electric permittivity (**a**) and loss tangent (**b**) as a function of temperature, measured at a frequency of 1 kHz, on heating processes for Ba_0.75_Ca_0.25_TiO_3_ ceramics sintered at various temperatures *T*_S_.

In order to investigate the effect of the final sintering temperature on the dielectric parameters of Ba_0.75_Ca_0.25_TiO_3_ ceramics, measurements of the temperature changes in electric permittivity (ε′) and the loss tangent (tan δ) were carried out for all five samples. Figure 3 shows such dependences obtained at a frequency of the measuring field equal to 1 kHz.

The maximum value of electric permittivity (ε′_max_) gradually increases with the rise in sintering temperature (Table 3). This trend persists until *T*_S_ = 1400 °C. At *T*_S_ = 1450 °C, a sharp decrease in ε′_max_ is observed. Such an effect has been observed, among others, for BaTiO_3_ ceramics [60] and PBZT 37/70/30 ceramics [61]. This phenomenon can be explained based on the defect model [62], which assumes that the factor suppressing the formation of the domain structure is crystal lattice defects, which tend to localize at grain boundaries. As the grain size increases, the contribution of grain boundaries to the sample volume decreases, leading to a reduction in the number of defects—allowing the domain structure to grow more freely [62]. Moreover, the increase in grain size leads to a decrease in the thickness of the relatively more insulating grain boundary layer [63]. As a result of these processes, an increase in electric permittivity is observed. Therefore, electric permittivity increases with the increase in grain size. The described trend breaks down for BCT ceramics sintered at 1450 °C, where the electric permittivity decreases across the entire measured temperature range. This is due to significant and rapid grain growth; the average grain size for this material is 5.6 μm, which is over three times larger than the average grain size of ceramics sintered at 1400 °C (1.6 μm, see Table 2). It is well known that in ceramics made from barium titanate, dielectric properties, particularly the value of the electric permittivity, strongly depend on grain size, and this relationship is not straightforward. Specifically, the authors of studies [60,64] found that in BaTiO_3_ ceramics, the optimal grain size for achieving the highest dielectric constant is 0.8 μm. Further reduction in grain size leads to a decrease in electric permittivity, which can be explained by the increased contribution of non-ferroelectric grain boundaries to the overall volume of the sample [65,66]. Increasing the grain size also leads to a decrease in the dielectric constant. In BaTiO_3_ ceramics with grain sizes in the range of 0.8–50 μm, there is an inversely proportional relationship between grain size and the dielectric constant. According to Buessem [67], this phenomenon is associated with an increase in residual stress within individual grains, accompanied by an increase in tetragonal distortion. It is also worth mentioning that in other systems based on barium titanate, the influence of sintering temperature on the increase in the maximum value of electric permittivity is observed. Examples of such systems include BZT-BC 50/50 [53] ceramics and (Ba_0.85_Ca_0.15_)(Sn_x_Zr_0.1x_Ti_0.90_)O_3_ ceramics [68]. In the latter case, the maximum electric permittivity (*ε_max_*) reaches its peak value at a sintering temperature of *T*_S_ = 1450 °C.

In the context of decreasing dielectric permittivity, it is also important to note the reduction in the porosity of materials. As the temperature increases, the porosity of the materials changes from 16% for ceramics sintered at *T*_S_ = 1250 °C to 1.5% for ceramics sintered at 1450 °C. Such a significant change in porosity affects the value of dielectric permittivity, potentially increasing it by a significant factor, as demonstrated by the authors of the study [69]. The changing grain/particle sizes and the proportion of pores in the total volume of the sample shape the properties not only of electroceramic materials but also other materials. Examples include graphene substrates [70] or thermoelectric materials [71].

Changes in the final sintering temperature also affect the value of the maximum electric permittivity temperature, which can be associated with the phase transition temperature between the low-temperature ferroelectric phase and the paraelectric phase. As *T*_S_ increases, the Curie temperature (*T*_C_) decreases (Table 3).

Additionally, the maxima of dielectric permittivity observed in Figure 3 exhibit broadening, which appears to be more pronounced at lower sintering temperatures. To fully assess this phase transition broadening, an attempt was made to fit both the classical and modified Curie–Weiss laws to the experimental data. Since the 1/*ε*(*T*) dependence does not immediately become linear above the *T*_C_ temperature, the classical Curie–Weiss law is only applied above the *T*_dev_ temperature. In the temperature range between *T*_C_ and *T*_dev_, the modified Curie–Weiss law is used (1):

(1) $$\frac{1}{\varepsilon^{\prime }}-\frac{1}{\varepsilon_{max}^{\prime }}=\frac{{\left(T-T_{C}\right)}^{\gamma }}{\delta }$$

where

*ε*_max_—maximum value of electric permittivity;

*δ*—the Curie-like constant;

*γ*—the diffuseness parameter.

**Figure 4:**
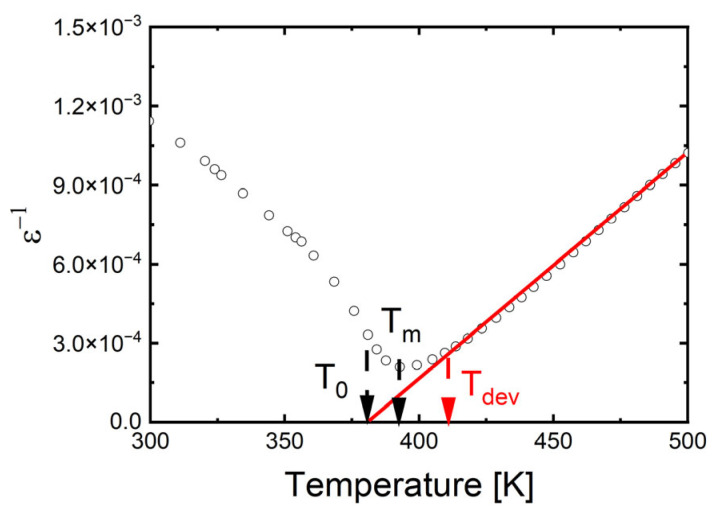
The reciprocal permittivity (1/ε) at 1 kHz as a function of temperature for Ba_0.75_Ca_0.25_TiO_3_ ceramics sintered at various temperatures, *T*_S_. Bullets indicate experimental data, red line is the fitting line.

A plot of ln(1/*ε* − 1/*ε*_max_) versus ln(*T* − *T*_C_) for the selected temperature (*T*_S_ = 1350 °C) is shown as an example in Figure 4. The obtained values of *T*_dev_, *T*_0_ and *C* are collected in Table 3. The value of the diffuseness parameter decreases significantly, indicating a reduction in the degree of phase transition diffuseness and an increase in the order of the crystal structure. The results obtained are in good correlation with SEM and XRD studies. The reduction in phase transition broadening due to an increase in sintering temperature was also observed in BZT ceramics [72]. In contrast, in BSN ceramics, raising the sintering temperature significantly increased the degree of phase transition broadening [68].

The effect of the temperature changes on the loss factor is shown in Figure 3b. In the case of materials sintered at temperatures of *T* = 1250 °C, *T* = 1300 °C and *T* = 1350 °C, only slight changes in the values of the loss tangent (tan δ) are noted in the temperature range of 325 K–400 K. A distinct loss peak, observed at the Curie temperature and indicating a phase transition, only appears for ceramics sintered at the two highest temperatures. All the presented characteristics exhibit a strong increase in losses above 450 K, which may be attributed to the increased mobility of charge carriers arising from defects or vacancies in the sample [51,73]. The value of the loss tangent at room temperature significantly decreases with the increase in sintering temperature (Table 3), which is extremely important from an application standpoint.

### 3.4. Thermal Analysis

**Figure 5:**
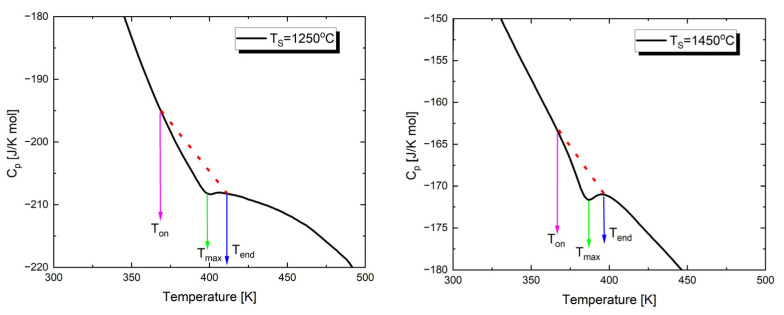
Temperature dependences of the specific heat capacity, C_p_, measured upon heating of Ba_0.75_Ca_0.25_TiO_3_ ceramics sintered at different temperatures.

As mentioned above, the temperature characteristics of dielectric permittivity show a peak associated with the transition from the low-temperature ferroelectric phase to the paraelectric phase. To investigate the effect of sintering temperature on the thermal parameters of this transition, the temperature dependence of specific heat was determined through calorimetric measurements. Figure 5 shows examples of the temperature dependencies of the specific heat capacity (C_p_) for Ba_0.75_Ca_0.25_TiO_3_ sintered at two extreme values of *T*_S_. The investigations were carried out during heating cycles.

In the presented characteristics, anomalies appear at temperatures (T_max_) corresponding to those of the dielectric permittivity peaks. These values are slightly lower than the Curie temperature (*T*_C_), which is a well-known behavior (Table 4) [74,75]. The temperatures corresponding to the onset (*T*_on_) and completion (*T*_end_) of the phase transition are also provided in Table 4. Additionally, Table 4 presents the values of the total enthalpy change, estimated from the area under the anomaly curve, using the background lines shown as dashed lines in Figure 5.

Analyzing the results presented in Table 4, it can be said that with the increase in sintering temperature, and consequently the development of a grain structure, the peak observed in the C_p_(T) dependencies becomes sharper. A similar trend was observed by the authors of study [76]; an increase in grain size leads to the sharpening of the anomaly. Additionally, it is worth noting that as the sintering temperature increases, the enthalpy decreases. These facts can be explained by the changing concentration of defects. Specifically, a high concentration of defects [77] and the deformation field caused by dislocations [78] can significantly influence the thermodynamic properties of the material near the phase transition. The high density of defects leads to an increase in heat capacity and a broadening of the heat capacity jump [79].

### 3.5. Pyroelectric and Thermally Stimulated Depolarization Currents

**Figure 6:**
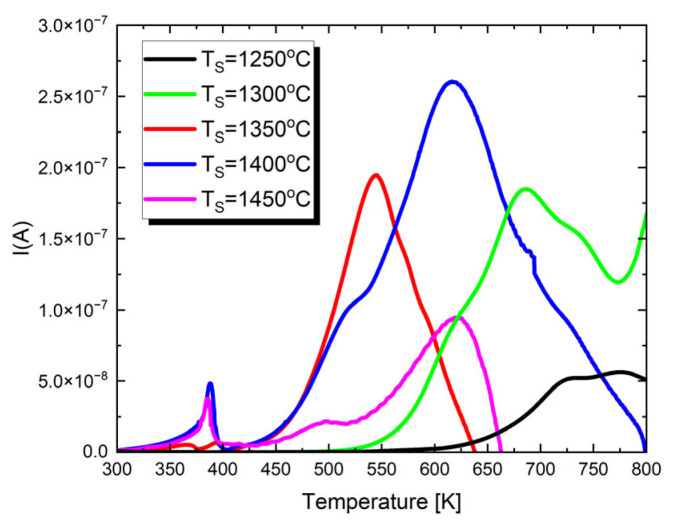
The temperature dependences of pyroelectric and thermally stimulated depolarization currents for Ba_0.75_Ca_0.25_TiO_3_ ceramics sintered at different temperatures.

To gain a deeper understanding of the dielectric properties and assess the potential applications of the studied materials, pyroelectric and thermally stimulated depolarization (TSD) current measurements were performed. The resulting characteristics are displayed in Figure 6.

In the presented I(T) characteristics, depending on the sintering temperature, one or two maxima appear. The first low-temperature maximum appears only in the case of materials sintered at *T* = 1300 °C, *T* = 1350 °C and *T* = 1400 °C. Its occurrence temperature coincides, within the measurement error, with the TC temperature. Therefore, this is the maximum of the pyroelectric current. The next maximum, visible for all discussed ceramic materials, is associated with the presence of strong thermally stimulated depolarization currents in the material. These currents reach their highest value for ceramics sintered at *T* = 1400 °C. Thermally stimulated depolarization currents are connected with the presence of material oxygen vacancies. The proposed mechanism for generating TSDCs in BaTiO_3_-based materials can be explained as follows [80]. During DC polarization at high temperatures, oxygen vacancies accumulate near the cathode and are depleted near the anode due to the blocking of ionic species by the metal electrodes. This results in a significant concentration gradient of $V_{o}^{∙∙}$. The spatial variation in the $V_{o}^{∙∙}$ concentration also induces gradients in electron and hole concentrations. After rapid cooling to lower temperatures, the DC field is removed, and the samples are short-circuited, leading to a metastable condition. The driving force for the depolarization of each species is the chemical potential created by these concentration gradients. Since electrons and holes move much faster than $V_{o}^{∙∙},$ their depolarization—producing an electronic current—occurs immediately after the samples are short-circuited. However, the polarized profile of $V_{o}^{∙∙}$ persists due to its much lower mobility. As the temperature increases, thermally activated depolarization of $V_{o}^{∙∙}$ takes place. This change in ionic polarization within the material generates image charges at the metal electrodes, producing the depolarization current. Therefore, the measured TSDC is governed by the relaxation of $V_{o}^{∙∙}$.

## 4. Conclusions

Ba_0.75_Ca_0.25_TiO_3_ ceramics were obtained by the solid-state synthesis method from simple oxides and carbonates. XRD studies confirmed the partial substitution of Ba^2+^ ions with Ca^2+^ ions in the tetragonal P4mm lattice characteristic of a pure BaTiO_3_ compound. However, only the ceramics sintered at *T*_S_ = 1400 °C and *T*_S_ = 1450 °C turned out to be single-phase. The sintering of samples at a temperature below *T* = 1400 °C caused a separation of small amounts of the Ca-rich orthorhombic phase. Microstructural analysis confirmed that higher sintering temperatures promote better grain boundary formation and a substantial increase in grain size. As the sintering temperature increases, the maximum electric permittivity (ε′_max_) rises up to *T* = 1400 °C but drops sharply at *T* = 1450 °C, which can be attributed to changes in the defect structure and domain formation. Simultaneously, an increase in the sintering temperature results in a reduction in the Curie temperature (*T*_C_), indicating changes in the phase transition behavior between the ferroelectric and paraelectric phases. Thermal analysis shows that higher sintering temperatures lead to a sharper peak in heat capacity, which is related to the development of the grain structure and a reduction in the number of defects. Additionally, pyroelectric and thermally stimulated depolarization (TSD) currents reach their highest values at *T* = 1400 °C due to the presence of oxygen vacancies in the material.

The lack of lead makes Ba_0.75_Ca_0.25_TiO_3_ ceramics a strong contender in fields where environmental concerns are paramount. The results presented in this paper serve as a starting point for further research aimed at improving the performance of this material, as well as enhancing its dielectric and piezoelectric properties and refining stability under various environmental conditions.

## Figures and Tables

**Table 1:** Structural parameters derived from Rietveld refinement procedure.

*T*_S_ [°C]	Crystal System	Space Group	Lattice Parameters [A]	Volume [A^3^]	Contrib. [wt. %]
1250	tetragonal	P4mm	a = b = 3.9679 c = 4.0068	63.08	94.6
	orthorhom.	Pbnm	a = 5.4548 b = 7.6615 c = 5.4083	226.02	5.4
1300	tetragonal	P4mm	a = b = 3.9646 c = 4.0032	62.92	96.1
	orthorhom.	Pbnm	a = 5.4628 b = 7.6767 c = 5.4163	227.14	3.9
1350	tetragonal	P4mm	a = b = 3.9599 c = 4.0012	62.74	97.7
	orthorhom.	Pbnm	a = 5.4945 b = 7.6855 c = 5.4208	228.9	2.3
1400	tetragonal	P4mm	a = b = 3.9565 c = 3.9999	62.62	100
1450	tetragonal	P4mm	a = b = 3.9564 c = 3.9997	62.60	100

**Table 2:** Density and average grain size of Ba_0.75_Ca_0.25_TiO_3_ ceramics sintered at different temperatures.

*T*_S_ [°C]	Average Grain Size [µm]	Experimental Density [g/cm³]
1250	-	4.47
1300	-	5.11
1350	0.70	5.41
1400	1.60	5.67
1450	5.4	5.47

**Table 3:** The influence of the sintering temperature (*T*_S_) on the dielectric parameters of Ba_0.75_Ca_0.25_TiO_3_ ceramics measured at a frequency of f = 1 kHz.

*T*_S_ [°C]	*T*_C_ [K]	*ε*_RT_	*ε*_max_	tgδ_RT_	tgδ_TC_	*T*_0_ [K]	*C* [K]	*T*_dev_ [K]	*γ*
1250	397.49	922.4	2345.712	0.119	0.016	373	5.9 × 10^4^	421	1.85
1300	395.01	1075.9	3450.791	0.016	0.009	376	1.2 × 10^5^	418	1.71
1350	392.09	839.9	4799.394	0.012	0.011	380	1.5 × 10^5^	415	1.60
1400	387.40	657.9	6772.963	0.007	0.014	364	1.1 × 10^5^	406	1.31
1450	385.39	660.6	5492.813	0.013	0.008	375	1.1 × 10^5^	407	1.26

**Table 4:** The influence of sintering temperature (T_S_) on the parameters of the FE-PE phase transition in Ba_0.75_Ca_0.25_TiO_3_ ceramics.

*T*_S_ [°C]	ΔH [J/g]	*T*_on_ [K]	*T*_end_ [K]	*T*_max_ [K]
1250	0.42	362.5	412.0	395.8
1300	0.37	368.9	414.8	392.0
1350	0.27	371.7	412.1	388.2
1400	0.26	362.5	404.7	384.3
1450	0.32	369.7	397.5	383.6

## Data Availability

The original contributions presented in the study are included in the article, further inquiries can be directed to the corresponding author.
